# Intrinsic factors associated with return to sport after anterior cruciate ligament reconstruction: A systematic review

**DOI:** 10.4102/sajp.v71i1.230

**Published:** 2015-06-11

**Authors:** Cheryl A. Ross, Amanda Clifford, Quinette A. Louw

**Affiliations:** 1 Department of Physiotherapy, Stellenbosch University, South Africa; 2 Department of Clinical Therapies, University of Limerick, Ireland

## Abstract

**Objectives:**

The anterior cruciate ligament is the most commonly injured ligament in the knee, with an average of only 64% of affected athletes returning to their pre-injury level of sport. Intrinsic factors associated with an increased likelihood of return to sport may be addressed during rehabilitation to improve the outcome of the reconstruction. The objectives of this review were to systematically appraise publications from six electronic databases describing intrinsic factors that may be associated with return to sport after anterior cruciate ligament reconstruction.

**Methods:**

The Preferred Reporting Items for Systematic Reviews and Meta-Analyses (PRISMA) guidelines were followed. Methodological quality appraisal was performed according to the Critical Appraisal Skills Programme for cohort studies. We performed a descriptive synthesis of the findings that associated intrinsic factors with return to sport.

**Results:**

Ten studies were included in the review. The findings show that fear of re-injury is a common reason for not returning to participation in sport. Younger patients may be more likely to return to sport, but findings regarding gender were equivocal, with male competitive athletes appearing to be more likely to return to sport than their female counterparts. Good knee function is not always associated with a higher likelihood to return to sport.

**Conclusion:**

Fear of re-injury and age should be considered in the management of sports participants after anterior cruciate ligament reconstruction.

## Introduction

The anterior cruciate ligament (ACL) is the most commonly injured ligament in the knee, resulting in devastating effects for the athlete (Ardern *et al.* 2011). Loss of knee stability may impair activity levels and, for many, have psychological and social implications of questionable return to sport (RTS) (Grindem *et al.* 2012). Appropriate management of ACL injuries is important to facilitate RTS. Conservative management is indicated in athletes not involved in pivoting sports or for those returning to a low level of physical activity (Grindem *et al.* 2012). However, surgical reconstruction of the ACL is required when conservative management has failed or for patients for whom RTS would be impossible with an unstable knee (Smith *et al.* 2004). Irrespective of the type of management, RTS after this common injury remains challenging (Grindem *et al.* 2012). Results of reviewed studies indicate that, on average, 81% of athletes have returned to some form of sports participation after 12 months and 55% have returned to competitive sport (Ardern *et al.* 2014).

Factors that indicate a likelihood of returning to sport are therefore important. Clinicians and coaches can access these factors and intervene to optimise an athlete’s chances of returning to sport. Extrinsic factors (originating outside the body), such as surgical procedure, rehabilitation protocols, sporting equipment and sport-specific coaching, may influence RTS. Rehabilitation before and after ACL surgery is imperative to facilitate timely and safe RTS (Cascio, Culp & Cosgarea 2004). To contribute to RTS, exercises such as running, strength training, proprioception and low-intensity sport can commence from 4–5 months after surgery, with sport-specific drills and moderate-intensity sport commencing at 6 months (Cascio *et al.* 2004; Petersen & Zantop 2013). Extrinsic factors are influenced by many personal and contextual factors and have been investigated by numerous studies (Engelman *et al.* 2014; Kim, Seon & Jo 2013; Saka 2014).

Intrinsic factors, which are inherent to the athlete, include age, gender, height and body weight (expressed as the body mass index [BMI]), muscle strength, flexibility, level of motivation to comply with rehabilitation, fear of re-injury, associated injuries to the knee or other lower limb joints, joint integrity on injury, and previous injury to or tearing of the ACL. It is unclear whether these intrinsic factors relate to RTS post ACL reconstruction. Age and gender are not modifiable, but can assist clinicians in planning the duration (and therefore associated costs) and structure of the rehabilitation programme. Knowing which, if any, modifiable intrinsic factors influence RTS will enable pro-active planning to ensure an athlete’s timely and safe RTS.

Two published systematic reviews investigated variables associated with RTS, but addressed broad questions. Ardern *et al.* (2014) aimed to update the RTS rate of a previous review, with a secondary aim to investigate physical and contextual factors associated with RTS. Czuppon *et al.* (2014) appraised the risk of bias across studies of various designs and described variables associated with RTS. Both reviews included studies of considerable heterogeneity with respect to study design, evidence levels, samples and aims.

The objective of our review is thus to systematically appraise all evidence for intrinsic factors exclusively and their association with RTS participation at the pre-injury level. RTS at the pre-injury level is considered an indicator of the success of ACL reconstruction for both competitive and recreational-level athletes (Lee, Karim & Chang 2008). This review will offer clinicians, patients, coaches and sports administrators a succinct evidence synthesis of intrinsic factors related to RTS to facilitate evidence-based management.

## Methods

This systematic review was conducted according to guidelines by Sterk and Rabe (2004) using the Preferred Reporting Items for Systematic Reviews and Meta-Analyses guidelines (PRISMA 2009). Cohort, case–control and cross-sectional studies published as peer-reviewed journal publications in English, French or German were considered. Publications that included either male or female participants (or a mixed-gender group) from 13 years and older (adolescents and adults) and who participated in physical activity (recreational or competitive sport) at least twice a week before sustaining an ACL injury were considered for review. Studies reporting on participants who required surgery to reconstruct the ACL using all graft types (hamstring or patellar tendon autograft, or allograft) were considered. All studies had to report on return to the samesport, at either the same or a lower intensity level. Intrinsic factors included, but were not limited to, age, gender, strength of the quadriceps muscle, fear of re-injury, leg dominance, BMI and degree of ACL laxity pre-operatively.

### Search strategy

The Stellenbosch University online library was used to search the electronic databases CINAHL, PubMED, Scopus, SPORTDiscus, Google Scholar and ScienceDirect. These databases are often used to search for literature pertaining to health-related systematic reviews (Wright *et al.* 2007). All selected databases were searched from inception until July 2014. Two searches were performed. ‘Anterior cruciate ligament’ was used as a standalone keyword for all search strategies. In the first search, combinations of keywords including [post surgery] AND [outcomes] AND [predictors] AND [physiotherapy OR physical therapy] AND [return to sport] were added to develop an appropriate search string. Medical subject heading (MeSH) terms were used in PubMED. A second, more refined search to find additional publications was performed by composing a more precise search string. Here, the intrinsic factors [age] OR [dominance] OR [muscle strength] OR [BMI] OR [body weight] OR [laxity] OR [gender] OR [fear] OR [activity level] were added independently as keywords to three databases, thus yielding the most relevant hits. Pearling of reference lists of included studies was performed.

### Study selection

One researcher screened the titles and abstracts of all initial hits. Two researchers (one not a listed author) independently screened all potential full-text papers, according to the aforementioned study criteria. To ensure consistency between approaches, a checklist for eligibility was developed. This checklist contained all eligibility criteria as described. Discrepancies between researchers’ views on or interpretation of the criteria were discussed until consensus was reached. A third researcher (a listed author) was consulted when deemed necessary.

### Methodological quality appraisal

The methodological quality of each study was appraised by one researcher using the Critical Appraisal Skills Programme (CASP) for Cohort studies (Public Health Resource Unit, NHS, United Kingdom). No randomised controlled trials were considered for inclusion, therefore eliminating the use of the CONSORT statement, which is a validated tool. CASP has separate scales for specific study designs; this scale assesses cohort studies only and was therefore appropriate for this review. As this tool can be used as either a checklist or a scoring system, it facilitates simple and reliable scoring. The critical appraisal tool comprised 12 criteria to which a ‘yes’, ’no’ or ’can’t tell’ response was assigned and justified. Two of the criteria did not yield the specified responses and were thus rephrased. (The original criteria, ’What are the results of the study?’ and ’How precise were the results?’, were rephrased to ‘Are the results clearly described?’ and ‘Have the probability values been reported?’, respectively.) All positive responses were tallied and a score was assigned for each study. The maximum possible score was a total of 12 points. From the selected studies, one was randomly chosen for appraisal by a second researcher and discrepancies in scores were discussed.

### Data extraction

Data extracted from each study were summarised using a customised data extraction spreadsheet. Information about the sample demographics, sample size, intrinsic factors (as defined earlier), type of sport, time from surgery to study assessment, level of sports participation, statistical procedures, findings and limitations of each study were extracted. The demographic variables included age and gender. A second researcher extracted the data of two randomly selected studies to ascertain the accuracy of data extraction.

### Data analysis or synthesis

A meta-analysis was not possible owing to the variations in study outcomes. There were also marked differences between statistical analysis procedures, intrinsic factors, and the type of data reported. For this reason, a descriptive synthesis of the findings was conducted. Information was tabulated to compare the findings of eligible studies. Odds ratios and 95% confidence intervals were calculated by means of a 2 × 2 table calculator for the five studies investigating the association of gender with RTS (Ardern *et al.* 2011,2013; Kvist *et al.* 2005; Lentz *et al.* 2012; Smith *et al.* 2004). This was repeated for the study by Osti *et al.* (2011), investigating the association of age with RTS, and two studies investigating knee function with RTS (Lee *et al.* 2008; Smith *et al.* 2004). A subgroup analysis of activity level with the associated intrinsic factors was performed, as the studies reviewed included a range of activity levels (from recreational to competitive).

## Results

**FIGURE 1:**
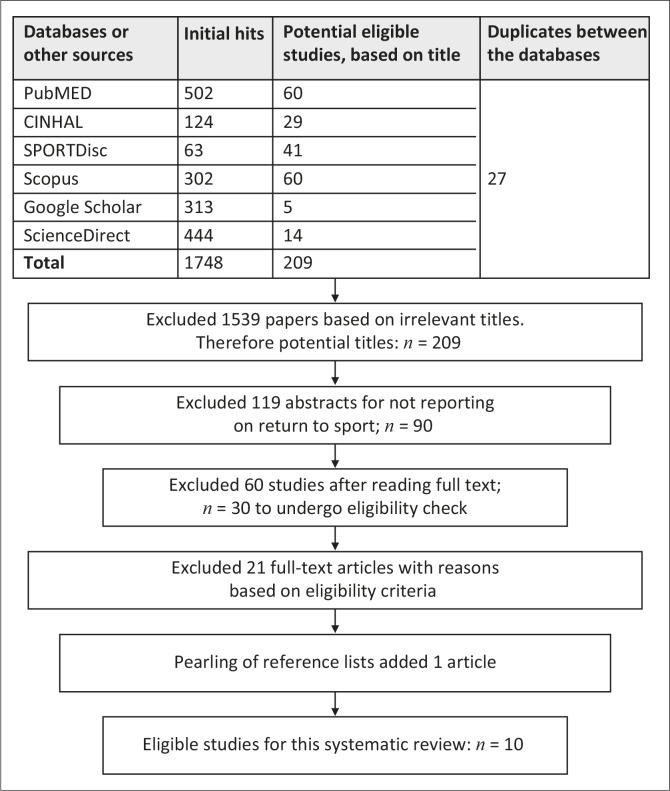
Flow diagram of search stratssegy to determine final sample for review.

The search strategy yielded 10 papers that met the described inclusion criteria, as shown in Figure 1.

### Critical appraisal of study quality

A median appraisal score of 10 (range: 8–11) was obtained after critical appraisal of study quality. The findings of the methodological appraisal, with the reasons for the negative scoring per criterion, are described in Table 1.

**TABLE 1:** Findings of critical appraisal of methodological quality.

Criterion	Ardern *et al.* (2011)	Ardern *et al.* (2013)	Devgan *et al.* (2011)	Gobbi and Francisco (2006)	Kvist *et al.* (2005)	Lee *et al.* (2008)	Lentz *et al.* (2012)	Osti *et al.* (2011)	Smith *et al.* (2004)	Tripp *et al.* (2007)
1. Clear aim	+	+	+	+	+	+	+	+	+	+
2. Appropriate method	+	+	+	+	+	+	+	+	+	+
3. Acceptable sampling	+	+	+	+	+	+	+	–^a^	+	+
4.										
Exposure accurately measured	+	+	+	+	+	+	+	+	+	–^e^
5.										
Outcome accurately measured	+	+	–^d^	+	+	+	+	+	+	+
6.										
Identification of confounding	+	+	–^d^	–^d^	+	+	+	+	+	+
7.										
Follow-up sufficiently long and complete	–^g^	–^g^	+	–^g^	–^g^	–^g^	–^h^	+	+	–^h^
8.										
Results clearly described	+	+	+	+	+	+	+	+	+	+
9.										
Reporting of probability values	+	+	+	+	+	+	+	+	+	+
10.										
Do you believe the results?	+	+	+	+	+	+	+	–^b/c/f^	+	+
11.										
Results applicable to local population	–^c^	–^c^	+	+	+	+	–^c^	–^c/f^	–^c/f^	–^c^
12.										
Do the results fit with other evidence?	+	+	+	+	+	+	+	–^d^	+	+
Score	10	10	10	10	11	11	10	8	11	9

Note: Please see the full reference list of the article, Ross, C.A., Clifford, A. & Louw, Q.A., 2015, ‘Intrinsic factors associated with return to sport after anterior cruciate ligament reconstruction: A systematic review’, *South African Journal of Physiotherapy* 71(1), Art. #230, 8 pages. http://dx.doi.org/10.4102/sajp.v71i1.230, for more information.

Reasons for negative score: ^a^, Sample bias or not described; ^b^, Confounding factors not taken into account; ^c^, Selection bias; ^d^, Not reported; ^e^, Patient not blinded/masked to purpose of study, thus intrinsic factor; ^f^, Small sample size; ^g^, Loss to follow-up; ^h^, No longitudinal follow-up.

### Study sample description

Of the studies reviewed, four had a similar number of male and female participants and six had approximately twice as many male as female participants (Table 2). Age ranged from 14–62 years, with an average age of 26.2 years in all the studies, except the one by Osti *et al.* (2011) in which separate age groups were investigated. The competitive level, sample size and time from surgery to follow-up in each study, including the RTS at both pre-injury and lower activity level, are presented in Table 2. Competitive-level athletes include athletes competing at a national or provisional level. Studies on competitive-level athletes showed a tendency towards a lower RTS rate. Time from injury to follow-up ranged from 1 year (the earliest possible return to competitive or pivoting sports) to 5 years (illustrating the sustainability of the reconstruction). The rate of RTS did not favour an earlier or later follow-up period. Athletes participated mainly in the following sports: soccer (Ardern *et al.* 2011, 2013; Osti *et al.* 2011; Tripp *et al.* 2007), basketball (Ardern *et al.* 2011, 2013; Osti *et al.* 2011; Tripp *et al.* 2007), skiing (Osti *et al.* 2011; Tripp *et al.* 2007), hockey (Osti *et al.* 2011), motocross (Tripp *et al.* 2007), netball (Ardern *et al.* 2011, 2013), athletics, martial arts and cricket (Devgan *et al.* 2011).

**TABLE 2:** Sample description for each study included in the systematic review.

Reference	Percentage returned to the same sport and activity level (%)	Percentage returned to modified sport or activity level (%)	Activity level	Sample size	Time from surgery to follow-up
Ardern *et al.* (2011)	33%	33%	Competitive	503	1 year
Ardern *et al.* (2013)	31%	Not reported	71% Competitive; 29% Recreational	178	1 year
Devgan *et al.* (2011)	46%	37.5%	Competitive – district, state and national	48	5 years
Gobbi and Francisco (2006)	65%	24%	All levels	100	3, 6, 12 and 24 months
Kvist *et al.* (2005)	53%	45%	All levels; 67% contact sport	62	3–4 years
Lee *et al.* (2008)	62%	Not reported	67% recreation participation twice a week; 33% competitive, including 3% national	64	5 years
Lentz *et al.* (2011)	55%	36%	All levels	94	1 year
Osti *et al.* (2009)	60% and 90%	35% and 5%	All levels and type of sports	40	2 years
Smith *et al.* (2004)	42%	19%	Competitive – elite	77	43 months
Tripp *et al.* (2007)	*Mean* score = 7.3 out of 10, *Standard Deviation* = 2.7 with higher values indicating more accomplished return	Recreational	49	1 year	

Note: Please see the full reference list of the article, Ross, C.A., Clifford, A. & Louw, Q.A., 2015, ‘Intrinsic factors associated with return to sport after anterior cruciate ligament reconstruction: A systematic review’, *South African Journal of Physiotherapy* 71(1), Art. #230, 8 pages. http://dx.doi.org/10.4102/sajp.v71i1.230, for more information.

### Study design, aims and outcomes

The studies included were cohort studies, prospectively following up athletes who underwent an ACL reconstruction. These studies included subjects with associated cartilage damage or meniscus repair, but excluded subjects if multiple ligament reconstruction or revision took place. All studies aimed to determine the factors associated with return or non-return to pre-injury activity level with a follow-up time of between 12 months and 5 years. Gender, age, muscle strength, knee ligament laxity, fear of re-injury, and knee function were described amongst the studies. There were no studies associating BMI and leg dominance with RTS post ACL reconstruction.

### The association of fear of re-injury with return to sport

Tripp *et al.* (2007) analysed whether fear of re-injury, negative affect (mood) or catastrophisation predicted RTS. They found that a high level of fear of re-injury was a significant predictor of not returning to sport (*P* = 0.01). Similarly, Ardern *et al.* (2013) investigated whether psychological responses pre-operatively and at 4 months post-operatively predicted RTS at 12 months. The findings indicated that psychological responses predicted RTS pre-operatively (*P* < 0.001). However, the optimal prediction of RTS was at 4 months according to the Anterior Cruciate Ligament/Return to Sport After Injury (ACL–RSI) scale. The Tampa Scale of Kinesiophobia (TSK) and Sport Rehabilitation Locus of Control (SRLC) scale were also predictive in the reduced model (*P* < 0.001). Unfortunately, the percentages of athletes who returned to sport and those not returning owing to fear of re-injury were not mentioned and thus prevented comparison between these two studies and others describing fear of re-injury.

Kvist *et al.* (2005) used the TSK to quantify fear of re-injury. Participants who did not return to sport scored higher on the TSK, indicating more fear of re-injury (*P* = 0.01). In addition, four other studies (Devgan *et al.* 2011; Gobbi & Francisco 2006; Lee *et al.* 2008; Lentz *et al.* 2012) also investigated fear of re-injury in relation to RTS. Across these studies, 141 athletes did not return to pre-injury sport, with an average of 35% (49 athletes) citing fear of re-injury as the reason. The percentages of athletes citing fear of re-injury in each study are displayed in Table 3.

**TABLE 3:** Summary of studies investigating fear as the reason for not returning to their previous level of sport.

Reference	Total assessed for return to sport	Athletes not returning to previous level of sport	Athletes citing fear as the reason for non-return	
			*n*	%
Devgan *et al.* (2011)^a^	40	18	12	67
Gobbi and Francisco (2006)^b^	100	35	26	
Kvist *et al.* (2006)^b^	62	29	7	24
Lee *et al.* (2008)^b^	45	17	9	53
Lentz *et al.* (2011)^b^	94	42	19	45
Total	341	141	49	35

Note: Please see the full reference list of the article, Ross, C.A., Clifford, A. & Louw, Q.A., 2015, ‘Intrinsic factors associated with return to sport after anterior cruciate ligament reconstruction: A systematic review’, *South African Journal of Physiotherapy* 71(1), Art. #230, 8 pages. http://dx.doi.org/10.4102/sajp.v71i1.230, for more information.

^a^, Competitive-level athletes; ^b^, Competitive and recreational athletes combined.

### The association of age with return to sport

Osti *et al.* (2011) investigated the association of age with RTS. They separated participants into two distinct age groups (< 30 years and > 50 years), with 20 athletes in each group. The athletes’ activity level at follow-up was compared with their pre-injury activity level, as older athletes generally participate at a lower intensity level than younger athletes. A significant difference was found: 90% of athletes in the younger group returned to sport, compared with a 60% return in the older age group. The level of sporting activity differed between age groups; older participants’ lower level of sporting activity pre-operatively was considered in analysis of RTS. The findings indicated that younger athletes were more likely to return to the pre-injury level of sport (odds ratio = 6; Confidence intervals [CI] = 1.08–33.28). Two studies (Ardern *et al.* 2013; Lentz *et al.* 2012) compared the mean age of the non-return group to those returning to pre-injury level of sport and found no significant difference (*P* = 0.066 and *P* = 0.6, respectively; α = 0.05). However, the samples were not separated into two age groups; therefore, findings could not be compared with those in the aforementioned study.

### The association of gender with return to sport

Five studies (Ardern *et al.* 2011, 2013; Kvist *et al.* 2005; Lentz *et al.* 2012; Smith *et al.* 2004) examined the association of gender with RTS at the pre-injury activity level. Odds ratios were calculated to indicate whether gender is associated with RTS. The results are displayed in Table 4.

**TABLE 4:** Association of gender with return to sport.

Reference	Study sample	Odds ratio	95**%** Confidence intervals (CI)	Significant association
Ardern *et al.* (2011)^a^	340 male and 163 female participants	1.70	1.12–2.57	Males significantly more likely to return to sport
Ardern *et al.* (2013)^b^	122 male and 56 female participants	1.34	0.71–2.73	No significant difference (CI not significant)
Kvist *et al.* (2005)^b^	34 male and 28 female participants	0.75	0.27–2.05	No significant difference
Lentz *et al.* (2011)^b^	60 male and 34 female participants	0.97	0.41–2.25	No significant difference
Smith *et al.* (2004)^a^	37 male and 40 female participants	1.50	0.55–4.08	No significant difference (CI not significant)

Note: Please see the full reference list of the article, Ross, C.A., Clifford, A. & Louw, Q.A., 2015, ‘Intrinsic factors associated with return to sport after anterior cruciate ligament reconstruction: A systematic review’, *South African Journal of Physiotherapy* 71(1), Art. #230, 8 pages. http://dx.doi.org/10.4102/sajp.v71i1.230, for more information.

^a^, Competitive-level athletes only; ^b^, Competitive and recreational athletes combined.

### The association of post-operative knee function/integrity with return to sport

Only one study (Gobbi & Francisco 2006) did not assess knee function at follow-up. The nine remaining studies are summarised in Table 5, with reference to the measurement tool used, the respective results and whether or not the outcome measure was found to be associated with RTS. This is important as these outcome measurement tools are frequently used to assess an athlete’s readiness for RTS. However, if they are not predictive of RTS, other reasons may influence athletes with good knee function scores to not return to sport. Lentz *et al.* (2012) questioned participants about the number of episodes of giving way or buckling of the knee since the surgery. They found significantly fewer episodes described by those returning to the pre-injury sport than by those who chose not to return to sport (*P* = 0.044). The strength of the quadriceps muscle was also tested in their study. Results showed that the quadriceps symmetry index was not significantly associated with RTS (*P* = 0.150); however, the normalised ratio of knee extensor torque to body weight did show a significant association (*P* = 0.050).

**TABLE 5:** Association of self-reported knee function/integrity with return to sport.

Author	Knee outcome measure	Reported findings	Association between knee function and RTS found?
Ardern *et al.* (2011)^a^	IKDC: (excellent compared to poor score)	Risk ratio, 1.5; 95% CI, 0.86–2.50	No
	IKDC (excellent compared to good score)	Risk ratio, 1.05; 95% CI, 0.81–1.40	
Ardern *et al.* (2013)^b^	Subjective IKDC	Subjective IKDC associated with RTS, *P* = 0.03	Yes
	Objective IKDC	Objective IKDC associated with RTS, *P* = 0.20	No
Devgan *et al.* (2011)^a^	Subjective and Objective IKDC/Lysholm scales	Objective IKDC associated with RTS, *P* = 0.004	Yes
		Subjective IKDC associated with RTS, *P* < 0.0001	
		Lysholm score associated with RTS, *P* < 0.0001	
Gobbi and Francisco (2006)^b^	Subjective and Objective IKDC, Noyes, Lysholm and Tegner scales	Subjective IKDC (*P* = 0.22); Objective IKDC (*P* = 0.38); Noyes (*P*= 0.053); Lysholm (*P* = 0.38); Tegner (*P* = 0.94)	No
	Marx activity scale	Athletes who returned to sport scored significantly higher than those who did not return (*P* < 0.001)	Yes
Kvist *et al.* (2005)^b^	Questionnaire KOOS	KOOS negatively correlated with TSK (*r* = –0.434, *P* < 0.05) and RTS correlated negatively with TSK (*P* = 0.01). It is therefore likely that KOOS will correlate with RTS.	Likely
Lee *et al.* (2012)^b^	Lysholm score/IKDC	IKDC Odds ratio, 0.22; 95% CI, 0.45–1.04	Yes
Lentz *et al.* (2012)^b^	Tegner scale, IKDC	Tegner (*P* < 0.001)	Yes
		IKDC (*P* < 0.001)	
Osti *et al.* (2011)^b^	IKDC	Participants who did not return to sport had more associated injuries, e.g. meniscus injuries	Yes
Smith *et al.* (2004)^a^	Questionnaire	Odds ratio, 3.4; 95% CI, 1.09–10.73	Yes

Note: Please see the full reference list of the article, Ross, C.A., Clifford, A. & Louw, Q.A., 2015, ‘Intrinsic factors associated with return to sport after anterior cruciate ligament reconstruction: A systematic review’, *South African Journal of Physiotherapy* 71(1), Art. #230, 8 pages. http://dx.doi.org/10.4102/sajp.v71i1.230, for more information.

CI, confidence interval; IKDC, International Knee Documentation Committee; KOOS, Knee Injury and Osteoarthritis Outcome Score; RTS, return to sport; TSK, Tampa Scale for Kinesiophobia.

^a^, Competitive-level athletes; ^b^, Athletes of all activity levels.

### Subgroup analysis of activity level with factors associated with return to sport

The current review considered studies that described various levels of sports participation, including competitive-level athletes (*n* = 3), all levels (*n* = 6) and recreational athletes (*n* = 1). A subgroup analysis was performed to identify factors showing a strong association with return to pre-injury level of sports participation in each subgroup. The results of the subgroup analysis are shown in Table 6.

**TABLE 6:** Subgroup analysis of studies that investigated activity level and factors showing strong association with return to sport.

Intrinsic factor	Competitive athletes	Recreational athletes	All levels of activity
Male gender	Ardern *et al.* (2011); Smith *et al.* (2004)	-	-
Less fear of re-injury	Devgan *et al.* (2011)	Tripp *et al.* (2007)	Ardern *et al.* (2013); Kvist *et al.* (2005); Lee *et al.* (2008); Lentz *et al.* (2011)
IKDC	Devgan *et al.* (2011)	-	Lee *et al.* 2008; Lentz *et al.* (2011)
Lysholm	Devgan *et al.* (2011)	-	-
Marx activity scale	-	-	Gobbi and Francisco (2006)
Tegner	-	-	Lentz *et al.* (2011)
Younger age	-	-	Osti *et al.* (2011)

Note: Please see the full reference list of the article, Ross, C.A., Clifford, A. & Louw, Q.A., 2015, ‘Intrinsic factors associated with return to sport after anterior cruciate ligament reconstruction: A systematic review’, *South African Journal of Physiotherapy* 71(1), Art. #230, 8 pages. http://dx.doi.org/10.4102/sajp.v71i1.230, for more information.

## Discussion

This systematic review highlights modifiable and non-modifiable intrinsic factors associated with RTS with participation at the same activity level as before the injury. We found that fear of re-injury is a common reason for athletes not returning to sport at all levels of participation. In three studies (Devgan *et al.* 2011; Lee *et al.* 2008; Lentz *et al.* 2012), approximately half the athletes cited fear as the reason for not returning to sport.

Fear of re-injury is a potentially modifiable factor. Physical problems such as impaired neuromotor control, poor proprioception or knee instability may be associated with fear of re-injury. Larmer *et al.* (2011) found that the fear of re-injury in participants who had recovered from an ankle ligament injury reduced after they practised performing the feared exercise. Following the ligament reconstruction and completion of the rehabilitation programme, it is assumed that the athlete may be confident to return to sport. However, our review findings illustrate that this assumption may not be true. It is thus important to increase awareness of the association between fear of re-injury and RTS amongst clinicians. Early identification and interventions aimed at reducing fear may be useful. Physical rehabilitation could be complemented with education and improving self-efficacy in an attempt to reduce fear of re-injury (Soderlund 2011).

Fear of re-injury may differ amongst recreational and competitive athletes. Re-injury or a long rehabilitation period negatively affects the competitive athlete, possibly diminishing the chances of returning to their position in the team (Kvist *et al.* 2005). To reduce fear of re-injury, the rehabilitation period should be optimised by motivating athletes to be compliant with their sport-specific exercises (Devgan *et al.* 2011). Tripp *et al.* (2007) suggest that fear of re-injury or movement is a form of avoidance behaviour evident in people with pain, which may further impair the neuromusculoskeletal system. Therefore, pain at the time of injury and surgery should be well managed to minimise this psychological component (Kvist *et al.* 2005). Ardern *et al.* (2013) indicate that psychological factors measured 4 months after surgery predicted RTS better than those measured pre-operatively. This indicates a temporal progression of fear of re-injury. It is unknown whether the fear exists pre-operatively or whether it develops through the rehabilitation process. These issues require further research.

The studies pertaining to fear of re-injury had methodological shortcomings. Firstly, male athletes were predominantly included and generalisation to female athletes is limited (Devgan *et al.* 2011; Gobbi & Francisco 2006; Lee *et al.* 2008; Lentz *et al.* 2012). In three of the studies we reviewed (Devgan *et al.* 2011, Gobbi & Francisco 2006; Kvist *et al.* 2005), questions regarding fear of re-injury were not directed to the entire sample, which may further compromise the generalisability of the study findings. In one study (Lentz *et al.* 2012), fear of re-injury was the most commonly cited reason for not returning to sport; however, in a multivariate analysis, the association was insignificant. Therefore, further research is required before conclusive findings can be drawn about the association.

We found only one study (Osti *et al.* 2011) that investigated whether age is related to RTS. The findings of that single study showed that younger athletes are more likely to return to sport; however, the power of the study was limited by a small sample size. Older athletes generally participate at a lower intensity level than younger athletes and therefore a subgroup analysis of activity level could not be performed. Younger athletes have more educational and occupational commitments; therefore this subgroup was excluded in the study by Lentz *et al.* (2012), who reported no association between age and RTS. When assessing RTS, a longer follow-up time may be required for older athletes (Soderlund 2011). Older athletes may also be less likely to return to sport owing to poorer knee function, muscle atrophy, proprioception deficits and pre-existing pathological conditions (Osti *et al.* 2011). Thus, it may be advisable for athletes to undergo ACL surgery as early as possible, if required. Whilst this systematic review considered adolescents only from the age of 13 years, a review by Vavken and Murray (2011) on ACL reconstruction in skeletally immature patients revealed good results of surgical treatment with minimal risk of growth disturbance. Therefore, a better RTS rate after ACL reconstruction is expected in younger athletes.

The findings regarding gender were inconsistent (Ardern *et al.* 2011, 2013; Lentz *et al.* 2012; Smith *et al.* 2004; Tripp *et al.* 2007). In one study (Ardern *et al.* 2011), with a large sample size, male athletes were significantly more likely to return to sport than female athletes. Owing to improved power the findings of the study by Ardern *et al.* (2011) are arguably more valid and generalisable than those of smaller studies. Thomee *et al.* (2007) indicated that following ACL reconstruction, male athletes had a significantly higher self-efficacy, which can be described as the judgement of one’s capability to perform difficult tasks (Soderlund 2011). A higher self-efficacy will be more advantageous in the competitive subgroup, where male athletes appear to be more likely to return to sport. Psychological factors such as fear and motivation may be different between male and female athletes and should be considered during rehabilitation. This association of gender with RTS warrants further research before valid conclusions can be made.

The intuitive assumption that good knee function relates to a better RTS rate may not always be true, as confirmed by the findings of Ardern *et al.* (2011) and Smith *et al.* (2004). However, the same scale for knee function was not used in all the reviewed studies, thereby limiting comparison between studies (Ardern *et al.* 2011; Devgan *et al.* 2011; Gobbi & Francisco 2006; Lee *et al.* 2008; Lentz *et al.* 2012). The reliability of the measurement tools, execution of tests and content of subjective questionnaires were stated in all studies. The Marx activity scale is positively associated with RTS; however, due to a large loss to follow-up, bias may have influenced the findings. Narducci *et al.* (2011) investigated the clinical utility of functional performance tests one year post ACL reconstruction in a systematic review, but did not find a test with construct or predictive validity for RTS. This may be a useful area for future research.

Our review included studies on physically active participants only. In this review, competitive athletes showed a generally lower RTS rate compared with studies that included athletes competing at all levels. In contrast, two studies (Devgan *et al.* 2011; Lee *et al.* 2008) reported a higher RTS rate amongst competitive athletes. Different motivational factors exist between competitive and recreational athletes. Competitive sport is more demanding and therefore recovery after injury may be associated with a lower rate of return (Smith *et al.* 2004). Tripp *et al.* (2007) suggested that recreational athletes who were concerned mainly with fitness might consider changing to another sport of similar intensity, but less threatening to the ACL. Focusing only on one subgroup of athletes prevents overestimating the RTS rate (Ardern *et al.* 2011), which may render the findings more reliable, albeit less generalisable.

The currently small evidence base, which excluded body weight and leg dominance, as well as a paucity of evidence regarding laxity and strength of the quadriceps muscle (Lentz *et al.* 2011), warrants future research. Lentz *et al.* (2012) found the ratio between quadriceps peak torque and body weight significantly associated with RTS, in contrast to other literature reporting inconsistent results of quadriceps strength on functional outcomes (Ross *et al.* 2002). Other factors may be associated with fear, including proprioception or neuromuscular control (Smith *et al.* 2004), pain (Kvist *et al.* 2005), gender and time from injury to surgery (Lee *et al.* 2008). These factors could be considered in future studies.

This review included studies assessing intrinsic factors at the time of follow-up when athletes are cleared for RTS, therefore demonstrating their association. However, it cannot be assumed that the same factors will be predictive of RTS if assessed prior to the athlete’s RTS. The review has a number of limitations. One limitation relates to the small evidence base, which is limited to 10 studies. As a meta-analysis was not possible owing to heterogeneity between studies, our review could have been subject to selection bias as titles were screened by one reviewer. A strength of the review is the sound methodological screening of studies to ensure that only high-quality studies were eligible for review. Furthermore, the focus of the research question was specific to intrinsic factors.

## Conclusion

The systematic review focused on intrinsic factors that may be associated with RTS after ACL reconstruction. The findings show that fear of re-injury is a common reason for not returning to participation in sport. Younger athletes may be more likely to return to sport, but findings regarding gender were equivocal, with male competitive athletes appearing more likely to return to sport than female athletes. Good knee function is not always associated with a higher likelihood of returning to sport. Fear of re-injury and age should be considered in the management of athletes after ACL reconstruction. Owing to the small, heterogeneous evidence base, further research is required.
